# Economic evaluation of participatory learning and action with women’s groups facilitated by Accredited Social Health Activists to improve birth outcomes in rural eastern India

**DOI:** 10.1186/s12962-017-0064-9

**Published:** 2017-03-21

**Authors:** Rajesh Kumar Sinha, Hassan Haghparast-Bidgoli, Prasanta Kishore Tripathy, Nirmala Nair, Rajkumar Gope, Shibanand Rath, Audrey Prost

**Affiliations:** 1 Ekjut, Ward Number 17, Plot 556B, Potka, District-West Singhbhum, PO-Chakradharpur, Jharkhand 833102 India; 2 UCL Institute for Global Health, 30 Guilford Street, London, WC1N 1EH UK

**Keywords:** Cost-effectiveness, Cluster randomised controlled trial, Participatory learning and action, Women’s groups, Neonatal mortality

## Abstract

**Background:**

Neonatal mortality remains unacceptably high in many low and middle-income countries, including India. A community mobilisation intervention using participatory learning and action with women’s groups facilitated by Accredited Social Health Activists (ASHAs) was conducted to improve maternal and newborn health. The intervention was evaluated through a cluster-randomised controlled trial conducted in Jharkhand and Odisha, eastern India. This aims to assess the cost-effectiveness this intervention.

**Methods:**

Costs were estimated from the provider’s perspective and calculated separately for the women’s group intervention and for activities to strengthen Village Health Sanitation and Nutrition Committees (VHNSC) conducted in all trial areas. Costs were estimated at 2017 prices and converted to US dollar (USD). The incremental cost-effectiveness ratio (ICER) was calculated with respect to a do-nothing alternative and compared with the WHO thresholds for cost-effective interventions. ICERs were calculated for cases of neonatal mortality and disability-adjusted life years (DALYs) averted.

**Results:**

The incremental cost of the intervention was USD 83 per averted DALY (USD 99 inclusive of VHSNC strengthening costs), and the incremental cost per newborn death averted was USD 2545 (USD 3046 inclusive of VHSNC strengthening costs). The intervention was highly cost-effective according to WHO threshold, as the cost per life year saved or DALY averted was less than India’s Gross Domestic Product (GDP) per capita. The robustness of the findings to assumptions was tested using a series of one-way sensitivity analyses. The sensitivity analysis does not change the conclusion that the intervention is highly cost-effective.

**Conclusion:**

Participatory learning and action with women’s groups facilitated by ASHAs was highly cost-effective to reduce neonatal mortality in rural settings with low literacy levels and high neonatal mortality rates. This approach could effectively complement facility-based care in India and can be scaled up in comparable high mortality settings.

## Background

The state of newborn health in India is of global importance, as India accounts for around a quarter of all neonatal deaths globally, with an estimated 779,000 out of 2.9 million neonatal deaths in 2012 [1, 2]. The national average neonatal mortality rate of 28 masks inequalities between richer and poorer states, as well as within states [3]. Although the proportion of women delivering with a skilled birth attendant has been increasing steadily in India, community interventions remain key to increasing demand for health services, birth preparedness as well as improving care for mothers and newborns at home [4].

A community based intervention called Participatory Learning Approach (PLA) approach with women’s groups reduced neo-natal mortality in the underserved areas of two eastern states of India, Jharkhand and Odisha [5], and the benefits accrued most to the most marginalised [6]. PLA with women’s groups gave similar results in terms of reducing neo-natal mortality in other studies conducted in Nepal, Bangladesh and Malawi [7–11]. A pooled analysis of the randomised control trials conducted in four different country sites India, Bangladesh, Malawi and Nepal also showed significant reduction in maternal mortality through PLA intervention [12].

Participatory Learning Approach (PLA) approach is a capacity building process in which women’s group members invite non group-members, adolescent girls, pregnant women, mothers, and men, frontline service providers for learning, planning, carrying out and evaluating activities in a participatory and sustained basis. Trained facilitators enable this community process. The approach is to engage communities in discussions on issues concerning them, build their understanding on the underlying causes and the cause and effect relationship, explore strengths and resources available and develop feasible strategies to overcome those issues. PLA approach has four phases. Phase one focusses on participatory identification and prioritization of maternal and child health problems besides sensitising community on the issue of ‘equity’ through picture cards and games. Phase two focusses on developing possible strategies for addressing the prioritised problems through different tools like storytelling and role plays. Games are employed to develop the understanding of the community members on cause and effect relationship, intermediate and underlying causes and possible prevention and management. This enables the community members to come up with the strategies together and collectively share the responsibilities among them to implement those strategies. Phase three is the action phase for the group where groups take actions according to the strategies they finalise with respective roles and responsibilities. Phase four is the stage where groups collectively evaluate the progress, learning and challenges throughout the PLA meeting cycle which they have gone through. This gives them idea of what worked well, what are their results, which are the areas where more effort is needed and how was the support from the other stakeholders.

From September 2010 to December 2012 (28 months), Ekjut, a charitable organisation registered under Societies Registration Act 1860 and working on maternal, newborn, child health and nutrition (MNCHN) in eastern India in collaboration with University College London–Institute for Global Health, UK, conducted a cluster-randomised controlled trial in five districts of Jharkhand and Odisha, eastern India, to assess the effect of a participatory learning and action (PLA) cycle with women’s groups facilitated by Accredited Social Health Activists (ASHAs) aiming to improve maternal and neonatal health. ASHAs are female community health workers incentivised under the National Health Mission (NHM) of India. They are primarily responsible for mobilising communities to increase demand for maternal and child health care services and promote institutional deliveries [13]. The objective of conducting this study was to see whether conducting PLA meetings by ASHAs with women’s group will achieve reduction in neo-natal mortality as were achieved previously in different trial settings.

It was found that, ASHA facilitated PLA women’s group intervention reduced neonatal mortality by 31 percent in the intervention area compared to the control area. The primary target groups for the PLA intervention were pregnant women and women of reproductive age from the indigenous (tribal) communities. These women were actively encouraged to participate in the women’s group meetings and join the women’s groups. As the focus was to actively target women from the marginalised communities, the results show that home visits by ASHAs in the intervention areas were more among the most marginalised groups compared to the less marginalised groups. Though home care practices for mothers and newborn were increased in both intervention and control areas, significant increase was found in the infants being wrapped in the intervention areas compared to the control areas. Similarly, newborn being placed to mother’s skin within 1 h was significantly increased in the intervention areas compared to the control areas. Proportion of institutional delivery was also found to be high in the intervention area compared to the control area. Since, throughout the intervention, focus was given to the women from the most marginalised community defined as those belonging to scheduled tribe community, being in the first and second lowest asset quintiles and unable to read and write, it was found that reduction in neo-natal mortality was the most among the most marginalised communities [14].

The aim of this paper is to measure the cost-effectiveness of participatory learning and action (PLA) with women’s group facilitated by ASHAs to improve birth outcome as compared to the control area. The analysis was done from the provider’s perspective, where project cost was analysed for reduction in neo-natal mortality using trial data.

## Methods

The trial was conducted in 30 purposively created geographical clusters of three underserved districts of Jharkhand (Ranchi, Khunti and Godda) and two districts of Odisha (Mayurbhanj and Rayagada), covering an estimated population of 156,519. 15 clusters were randomly allocated to receive the PLA intervention (estimated population 82,702), and 15 clusters to a control arm (estimated population 73,817) [14] These districts were selected because about half of the population of these districts belong to Scheduled Tribe^1^ community. However, in the study population, around 70% of the population belong to Scheduled Tribe community that have very high maternal and neonatal mortalities compared to the state and national average [14].

In the intervention clusters, 137 ASHAs were incentivised to conduct PLA meetings with women’s groups. Also, in both the intervention and control clusters, Ekjut organised monthly meetings with the village health sanitation and nutrition committees (VHSNCs) about their rights and entitlements. The details of PLA meetings and VHSNC meetings are described elsewhere [14]. One intervention cluster (estimated population 6203) was lost to follow up and the trial results have been presented comparing 14 intervention clusters (estimated population 76,499) and 15 control clusters (estimated population 73,817) [14]. The intervention reduced neonatal mortality by 31% (adjusted odds ratio [aOR] 0.69, 95% CI 0.53–0.89) during a 24 months’ intervention period (Jan 1, 2011, to Dec 31, 2012) [14]. We conducted a cost-effectiveness analysis comparing the women’s group intervention facilitated by ASHAs with current practice.

We conducted a cost-effectiveness analysis from the provider’s perspective. We included costs incurred by the organisation (Ekjut) implementing the intervention. Project costs were taken from the organisation’s accounting data. The accounting system was designed so that most costs could be identified separately for different cost centres. As these data are financial or accounting costs, they were converted to economic costs [15].

As suggested by Ramsey et al. [16], the time horizon was determined by the trial duration, i.e. 28 months from September 2010 to December 2012. This includes the start-up period for activities conducted before the intervention began and implementation period. The period between September 2010 and December 2010 was considered as ‘start-up’ period. During this period, we undertook preparatory exercises to start women’s groups and set up a monitoring system to collect data related to births and neonatal deaths.

We used a step-down costing methodology [17]. Costs from project accounts were fed into a customized tool created in MS Excel. The worksheets for entering data allocated costs to one of the following categories: staff, other recurring costs, capital, and joint costs. The costs were further divided into start-up and implementation costs, and between programme components (i.e. Women’s Group, VHSNC Strengthening), and research costs. Table 1 provides an overview of cost categorisations.

**Table 1:** Description of cost items and their categorisation

Cost categories	Description
Start-up costs	Identification and initial discussions with ASHAs; trainings with ASHAs; hiring support staff for women’s group intervention and monitoring staff; setting up the monitoring system; training of monitoring team; initial contacts with community members; printing picture cards; setting up the accounting system to collect cost data
Women’s groups costs	Incentives to 137 ASHAs for conducting women’s groups meetings; incentives paid to 15 co-facilitators who supported ASHAs in mobilising women and keeping records of the meetings; supervision costs for the women’s group intervention paid to five supervisors; time and other resource costs like transport and communication of staff members who supported the women’s group intervention
VHSNC strengthening costs	Incentives paid to five facilitators who conducted meetings with VHSNCs; supervision cost of VHSNC strengthening works paid to one supervisor for his time including other resource costs like transport, communication etc. and other costs required to support VHSNC strengthening works
Research costs	Incentives paid to community identifiers to collect information about births and newborn deaths in their catchment areas, incentives paid to 30 monitoring staff members who collected data on births and neonatal deaths; supervision cost of data collection work and quality checks paid to five supervisors, incentives paid to Data Inputters and Data Manager, time and other resource costs like transport, communication for other staff members who supported quantitative and qualitative data collection, data management and data analysis
Joint costs	Costs related to general administration, incentives paid and other related costs for accounting and administrative staff, time and resources of all staff involved in general administrative works, office rents, utility charges, maintenance cost, postage, general printing costs, bank charges against remittances, incentives paid for office assistance, security guards

Costs that could be easily identified with different programme components (women’s group, VHSNC strengthening and research costs) were allocated to them directly. Joint costs (for example general administration, office running costs and utility charges) were not easily identifiable with programme components and were initially put under joint costs. Joint costs were then allocated to the program components in proportion to their costs in relation to the total costs, in order to get the final total cost of the program components. For allocating staff time, monthly time sheets were completed after discussing with the concerned staff. Staff time was allocated to the above programme components and joint costs using completed time sheets. The same basis for allocation was taken for different costs items, including fuel, travel, communication and maintenance costs. Capital costs were converted into yearly expenditure with a depreciation of 10% on diminishing value. Start-up costs were capitalised over the project life cycle, assuming that these investments would have effects until the end of the project. As suggested by Gilson et al. [18], all costs related to newborn births and deaths surveillance were considered as research costs and were excluded from the cost analysis.

All costs were inflated to estimate their present value of 2107 price and converted to 2017 US dollar prices [USD1 = 68.07 Indian rupees (INR)] [19].

To examine the effect of variations in some uncertain variables on the incremental cost per DALY averted, we performed a series of one-way sensitivity analyses. This was done to check the robustness of our findings to assumptions. Table 2 describes the variables tested while performing these sensitivity analyses. These included the discount rate for both costs and outcomes, exchange rate, changing the number of neonatal deaths averted using the confidence interval for the odds ratio for the reduction in neonatal mortality from the parent trial, and changing the life-expectancy at birth in the calculation of DALY.

**Table 2:** Variables and values used in the sensitivity analyses

Variable	Lower limit	Base-case scenario	Upper limit	Reasoning
Changing discount rate (base case 3% both costs and outcomes)				WHO guide to cost-effectiveness analysis. Geneva: World Health Organization, 2003.
	Costs 0%, life-years 0%	3%	Costs 6%, life-years 3%	
Exchange rate (USD to INR)	50	68.07		Observed variation during the trial period
Number of neo-natal deaths averted	18	50	76	The upper and lower limits of the 95% confidence interval of odds ratio of reduction in neo-natal mortality
Life-expectancy at birth (years)	68.5^a^	86		Consistent with standard practice in economic assessment to take standard expected life expectancy. GBD-2010

^a^ Life expectancy at birth 2016 in India (http://www.indexmundi.com/india/life_expectancy_at_birth.html)

## Results

161 women’s groups were created in the intervention area. Each women’s group had undergone 33 meetings by the end of intervention period. 137 ASHAs facilitated these participatory learning and action meetings. Table 3 provides an overview of the intervention costs. The total unit cost for facilitating a women’s group was USD 24 and the total unit cost per women’s group was USD 790. The average intervention cost per person was USD 1.66 (USD 1.99 including VHSNC strengthening costs) and average intervention cost per livebirth was USD 35.31 (USD 42.28 including VHSNC strengthening costs).

**Table 3:** Overview of intervention cost

	Women’s groups	Women’s groups and VHSNC strengthening
Estimated population in the intervention	76,499	
Estimated number of live births in the intervention area	3603	
Total cost (INR)	8,660,561	10,368,255
Total cost (USD)	127,230	152,318
Average intervention cost per newborn child (USD)	35.31	42.28
Average intervention cost per person (USD)	1.66	1.99
Cost/women’s group meeting (USD) (33 meetings were conducted with each women’s group of total 161 groups)	24	
Cost per women’s group (USD) (161 women’s groups in the intervention clusters)	790	

*INR* Indian rupees, *USD* US dollar

The total intervention cost of ASHAs facilitating women’s group’s using participatory learning and action was USD 127,230 (USD 152,318 inclusive of VHSNC strengthening cost). During the implementation period (Jan 1, 2011 to Dec 31, 2012), we identified 3700 births (including 3,603 livebirths) in the intervention arm, and 3519 births (including 3439 livebirths) in the control arm. The neonatal mortality rate during the follow up period between 2011 and 2012 was 30 per 1000 livebirths in the intervention arm and 44 per 1000 livebirths in the control arm. We observed a 31% reduction in neonatal mortality (adjusted odds ratio [aOR] 0.69, 95% CI 0.53–0.89) in the intervention arm compared to the control arm [14]. After adjusting for the number of livebirths in the intervention and control arms, we found that the intervention averted 50 neonatal deaths in the intervention areas (refer Table 4).

**Table 4:** Estimate of neo-natal deaths averted

	Intervention arm	Control arm
Estimated number of live births in the intervention area	3603	3439
Neo-natal mortality rate (per 1000 live births)	30	44
Estimated neo-natal deaths (after taking estimated live births of intervention and control areas)	108	151
Estimated neo-natal deaths in the control area after taking same number of live births as in the intervention area		158
Neo-natal deaths averted (158 minus 108) in the intervention area	50	

For calculating DALY averted, disability weight for neo-natal death was taken of 1. The life expectancy of Japanese women [20] of 86 years [21] was taken as standard value. Discount rate for discounting future lives was taken of 3% and age of onset of neo-natal death was taken 0 year. Using these parameters, years of life lost due to premature deaths (YLLs) for one child was estimated to be 30.81 years. Hence, for averting 50 children’s deaths, the total years of life lost due to premature deaths (YLLs) averted or DALYs averted were estimated to be 1541 [22].

Table 5 provides an overview of the cost-effectiveness results. The cost of the intervention per DALY averted was USD 83/DALY (USD 99/DALY, inclusive of VHSNC strengthening cost) and the cost per newborn death averted was USD 2545 for the women’s group intervention only (USD 3046 inclusive of VHSNC strengthening cost) at 2017 prices. According to WHO, an intervention can be considered highly cost-effective if the cost per life year saved or DALY averted is less than the per capita Gross Domestic Product (GDP) of that country [23]. The women’s group intervention can therefore be considered as ‘highly cost-effective’ as it is much less than India’s per capita GDP in 2017 (USD 1830) [24].

**Table 5:** Overview of cost-effectiveness results

	Women’s groups	Women’s groups and VHSNC strengthening
Total cost (INR)	8,660,561	10,368,255
Total cost (USD)	127,230	152,318
Newborn deaths (ND) averted (difference in number of neonatal deaths between intervention and control areas)	50	
Life year saved per death averted	30.81	
Total life year saved or DALY averted	1541	
Cost per neonatal death averted (USD)	2545	3046
Cost/DALY averted (USD)	83	99

*INR* Indian rupees, *USD* US dollar

Table 6 describes the results of the sensitivity analyses. We found that the cost per DALY averted varied from USD 25/DALY (USD 30/DALY inclusive of VHSNC strengthening cost) to USD 273/DALY (USD 326/DALY inclusive of VHSNC strengthening cost) in response to changes in the uncertain variables. The variations in cost-effectiveness results were significant when the outcome was discounted at 6%, and when neonatal mortality reduction was set at the lower end of the confidence interval observed during the trial. The sensitivity analysis does not change the conclusion that the intervention is highly cost-effective.

**Table 6:** Results of sensitivity analyses

	Cost/DALY averted in women’s group intervention (cost/DALY averted including VHSNC strengthening activities)-in USD		
	Lower limit	Base-case scenario	Upper limit
Changing discount rate (base case 3% both costs and outcomes)			
Costs 0%, life-years 0%	25 (30)	83 (99)	
Costs 6%, life-years 3%			98 (118)
Exchange rate	134 (160)		–
Life expectancy at birth	104 (125)		–
Number of neonatal death averted	273 (326)		65 (77)

## Discussion

The women’s group PLA intervention facilitated by ASHAs was a highly cost-effective intervention to reduce neonatal mortality, according to WHO standards. In 2017, India’s expected GDP per capita would be USD 1830 and our estimate of cost life year saved (USD 83) falls well below this threshold. A comparison of ASHA-facilitated PLA women’s group intervention with similar PLA women’s group interventions implemented by Ekjut in India shows that this intervention was somewhat less cost-effective than previous ones. The cost per DALY of the previous two women’s group interventions led by Ekjut with its own trained and supported facilitators were USD 42 between 2005 and 2008 and USD 26 between 2008 and 2009 after inflating them to 2017 USD [5, 25]. However, the intervention was more cost-effective than other interventions with women’s groups practising participatory learning and action to improve maternal and newborn health in other parts of the world. The cost per DALY averted or life year saved using participatory women’s groups in Makwanpur district, Nepal, between 2000 and 2003 was USD 163 after inflating it to 2017 USD [7, 8]. Similarly, the cost per DALY averted for PLA with women’s group intervention under Perinatal Care Project in Bangladesh between 2009 and 2011 ranged from USD 263 to USD 469 after inflating them to 2017 USD [9]. Also, the cost per DALY averted for a similar intervention in rural Malawi between 2005 and 2009 was USD 140 (including both neonatal and maternal deaths averted) and MaiKhanda between 2008 and 2010 was USD 89 for community intervention (CI) after inflating them to 2017 USD [10, 11].

If the women’s group intervention was implemented on a larger scale, average costs would, in all likelihood, be reduced due to economies of scale. These might even outweigh the reduced effectiveness of interventions often documented in scale up programmes [26]. Also, costs may be further reduced if the coverage per district would increase because in the current trial, management and support structure created to support the intervention could have potentially served a larger geographical area and larger population. This could have also saved many more lives and hence could have lowered the cost per life year saved, without additional support/management cost. Training costs could also be further reduced as during the trial, a cascading training plan was used where support staff were centrally trained in four phases, followed by decentralised training of ASHAs in five phases. If the intervention was scaled up to a larger geographical area, a pool of trainers from different blocks (large administrative areas of 100,000 population) could be created to provide trainings to ASHAs directly in their respective blocks.

Ministry of Health and Family Welfare (MoHFW), Government of India (GoI) has decided to scale up ASHA facilitated PLA to reduce maternal and neonatal mortality in ten Indian states and issued government order in this regard to the NHM in all ten states. National Health System Resource Centre (NHSRC) working under MoHFW, GoI and Ekjut is providing technical support to all ten states’ NHM in scaling up PLA. NHM in Jharkhand has already implemented ASHA facilitated PLA on maternal and newborn care across the state of Jharkhand. However, for training of ASHAs, a slightly modified mechanism is being tried out. In the trial setting, Ekjut imparted trainings to all ASHAs from the intervention area and after that, ASHAs conducted PLA meetings with women’s group. In this scaling up model, instead of imparting PLA trainings directly to approximate 40,000 ASHAs working across the state, trainings will be imparted to approximate 2000 “ASHA facilitators” (supervisors) from the state. Each “ASHA facilitator” supports and supervises approximate 20 ASHAs from 20 villages and hamlets. These “ASHA facilitators” will be trained to conduct PLA meetings with women’s groups. They will then provide on-the-job trainings to the ASHAs they supervise. In the proposed scale up model, one “ASHA facilitator” will conduct a women’s group PLA meeting every month in ten villages together with the ASHAs from these villages and in the presence of ten other ASHAs from the neighbouring villages whom they supervise. This on the job, observational training will enable the visiting ASHAs to conduct similar meetings in their own villages. Every month, “ASHA facilitators” will rotate between their two groups of ten villages and conduct PLA meetings. This arrangement will reduce the costs of training substantially and enable faster scale up across the state. The reduction in start-up time for women’s group meetings will also cut a substantial amount of time, operational and management costs that would have been incurred if the ASHAs had been trained directly instead of through on-the-job training by “ASHA facilitators”. In Jharkhand, the scale up is being done in collaboration with the state’s National Health Mission; management and support structures are already in place, and the incremental costs for supporting PLA by the existing staff will be much less compared to what it would have been if a separate support structure had been created for this. A large scale controlled study has been planned and will be conducted by University College London–Institute for Global Health, UK. This will be done by prospective monitoring of births and deaths through a robust surveillance system. This evaluation will cover around ten million population to assess whether scaling up of ASHA facilitated PLA with women’s groups through this method is as effective as the previous ones in terms of reducing maternal and neo-natal deaths. It would also be wise to assess the cost-effectiveness of the revised scaling up model.

To scale-up PLA with women’s group through ASHAs across the country, we calculated the approximate scale-up costs. Four major cost components, which are important for scaling PLA with women’s groups through ASHAs are—trainings of ASHAs on PLA meeting cycle, printing of ASHA modules, printing of picture cards and incentives to ASHAs for organising PLA women’s group meetings. It was assumed that the current management and supervision mechanism already put in place to manage and supervise ASHAs’ work under National Health Mission (NHM) at the national and state levels will take care of ASHA facilitated PLA with women’s group program. Hence, no incremental costs related to management and supervision of this program were considered for assessing the total incremental costs of ASHA facilitated PLA with women’s groups. There are around 9,00,000 ASHAs working across the country [14]. Table 7 gives details of budgetary outlay for scaling up ASHA facilitated PLA. The total approximate budgetary outlay to support PLA with women’s group through ASHAs across the country would be around INR 378 crores. This is around 0.77% of the total health budget allocated for the year 2017–2018 and around 1.39% of the total budget allocated for National Health Programme (NHM) during the same year, which is primarily initiated to improve indicators like maternal mortality ratio (MMR), infant mortality rate (IMR), under-five mortality rate (U5MR) and total fertility rate (TFR) through different schemes. This shows that, ASHA facilitated PLA women’s group program can easily be scaled up by the government across India through a large network of ASHAs without substantial budgetary outlay. This will not only make community empowered and aware about maternal and newborn care practices but also lead to increase in uptake of existing maternal and child care programs being run under the umbrella of NHM. ASHAs are community health workers and have been given responsibilities of increasing uptake of ante-natal checkups, institutional deliveries, home visits and counselling, immunisation and disseminating information related to maternal and child care. Facilitating PLA with women’s groups will not only give ASHAs a platform to disseminate information related to maternal and child care but also improve their interpersonal skill and building community awareness and knowledge which lead to increase in demand of available services related to maternal and child care. This will make the job of the ASHAs easier and strengthen their relation with the community members.

**Table 7:** Scale up cost of ASHA facilitated PLA women’s group intervention across India. Source: CBGA (February 2017): what do the number tell? An analysis of Union Budget 2017–2018

Particulars	Amount (INR) per ASHA/ASHA facilitator	Total amount (INR in crores) for nine hundred thousand ASHAs
Training on PLA facilitation to ASHA facilitators (as 20 ASHAs are supervised by one ASHA facilitator, around 45,000 ASHA facilitators will be trained on different phases of PLA for 10 days at the rate of INR 1000 per day. These ASHA facilitators will provide support to nine hundred thousand ASHAs to conduct PLA with women’s groups)	10,000	45
Printing of PLA modules for ASHAs	500	45
Printing of picture cards for ASHAs (for each ASHA, 20 picture cards @ INR 10 per card will be printed)	200	18
Incentives to ASHAs for facilitating PLA meetings with women’s groups (Each ASHA will conduct 20 meetings with women’s group and will be incentivised @ INR 150 per meeting)	3000	270
Total budgetary outlay for scaling up ASHA facilitated PLA with women’s groups across India		378
Total budgetary allocation in health in India during the year 2017–2018 (% of total budgetary outlay for scaling up ASHA facilitated women’s group to total health budget during the year 2017–2018)		48,852.5 (0.77%)
Total budgetary outlay for NHM in India during the year 2017–2018 (% of total budgetary outlay for scaling up ASHA facilitated women’s group to total NHM budget during the year 2017–2018)		27,131.2 (1.39%)

Participatory learning and action with women’s groups facilitated by ASHAs is well suited to high mortality settings in India, particularly in underserved areas where access to health facilities and supply-side interventions is limited. PLA can only bring down avoidable deaths to some extent, due to changes in home care practices and community action for maternal and newborn health. However, to further reduce maternal and neonatal mortality rates, it would need to strengthen the public health system so that women and children can easily access quality health services. There is also evidence that marginalised mothers benefit the most from this approach [6, 22]. The approach works best if the coverage of a women’s group is about 450–700 population, and at least a third of the pregnant women participate in the women’s groups meetings [23]. PLA with women’s group intervention is recommended for those areas where marginalised population are living, where maternal and neonatal mortality rates are very high and where there is limited reach of government health services. In such places, this intervention is effective within limited resources, by cutting down the delays in identifying risks and seeking care, and in changing practices at home. Together with efforts to increase access to quality, facility-based maternal and newborn care, community interventions such as participatory learning and action with women’s groups can save lives.

## Conclusion

Participatory learning and action with women’s groups has been proven to reduce neonatal mortality in high-risk areas of India and is highly cost effective. There is substantial potential for replicating the intervention in other parts of India through Government systems. In India, the intervention can be taken up by ASHAs through regular government programmes in a cost-effective manner. The government can also use innovative models for scaling up participatory learning and action with women’s groups, thereby reducing the cost of scale up without compromising the quality of intervention. This would further improve the cost-effectiveness of the intervention so that government can use its limited resources efficiently.

## Abbreviations

ASHAs: Accredited Social Health Activists
VHSNCs: Village Health Sanitation and Nutrition Committees
USD: United States dollar
ICER: incremental cost-effectiveness ratio
WHO: World Health Organisation
DALYs: disability-adjusted life years
GDP: Gross Domestic Product
PLA: participatory learning and action
NHM: National Health Mission
aOR: adjusted odds ratio
INR: Indian rupees
LYS: life year saved
CHOICE: choosing intervention which are cost effective
GBD: global burden of disease

## Footnotes

1 Scheduled Tribe (ST) is an officially designated group recognised in the Constitution of India. The tribes under this group are historically disadvantaged indigenous people in India.
